# Current Therapeutic Approaches to Chronic Central Serous Chorioretinopathy

**DOI:** 10.4274/tjo.galenos.2018.49035

**Published:** 2019-02-28

**Authors:** Samet Gülkaş, Özlem Şahin

**Affiliations:** 1 Şanlıurfa Training and Research Hospital, Ophthalmology Clinic, Şanlıurfa, Turkey; 2 Marmara University Faculty of Medicine, Department of Ophthalmology, İstanbul, Turkey

**Keywords:** Central serous chorioretinopathy, subthreshold micropulse laser, anti-vascular endothelial growth factor, verteporfin photodynamic therapy

## Abstract

Central serous chorioretinopathy (CSCR) is the second most common maculopathy after diabetic maculopathy between the third and fifth decades of life. CSCR is characterized by serous neurosensory retinal detachment occasionally coexisting with retinal pigment epithelium (RPE) detachment. CSCR usually has good clinical prognosis, often resolving spontaneously within the first three months. However, some patients may have recurrent episodes and chronic disease. CSCR can cause permanent visual loss due to persistent neurosensory retinal detachment and RPE atrophy, especially in chronic cases. In recent years, verteporfin-photodynamic therapy applied with standard and low-dose/low-fluence protocols, anti-vascular endothelial growth factors, glucocorticoid antagonists, mineralocorticoid receptor antagonists, and subthreshold micropulse laser with varying parameters have been investigated as treatment options. In this review, we evaluated randomized and non-randomized case series conducted after 2000 that included at least 3 patients with chronic CSCR over 3 months in duration who were treated with current treatment options for chronic CSCR.

## Introduction

Central serous chorioretinopathy (CSCR) is characterized by serous neurosensory retinal detachment (NSD) accompanied by retinal pigment epithelium (RPE) detachment in some cases, and is the second most common maculopathy after diabetic maculopathy between the third and fifth decades of life.^1,2,3^ Clinically, CSCR has a good prognosis and usually resolves spontaneously within the first 3 months.^2,3^ However, approximately 5% of cases can become chronic.^1,4^ Refractory NSD, which can develop in chronic CSCR, may lead to photoreceptor damage, diffuse RPE changes, RPE atrophy, and subsequent permanent vision loss.^1,2,3^

**Figure 1:**
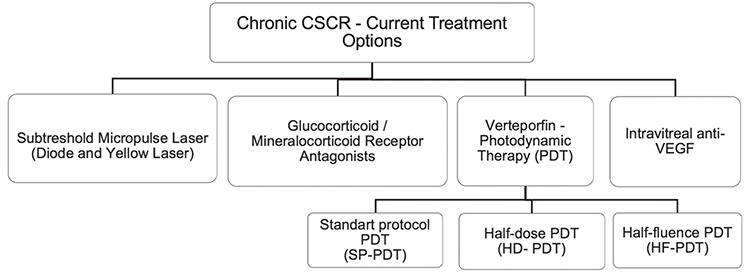
Current treatment options for chronic central serous chorioretinopathy

Studies on the subject have demonstrated that the two main factors involved in the pathogenesis of CSCR. The first is alterations in the autoregulatory mechanisms of choroidal circulation and the subsequent choroidal ischemia, and the second is irregularities in RPE pump function.^5,6,7^ Choroidal stasis, inflammation, and ischemia due to dysregulation of regulatory proteins (glucocorticoids, mineralocorticoids, epinephrine, norepinephrine) in the choroidal circulation leads to an increase in choroidal permeability.^7,8,9,10^ This hypothesis is corroborated by the presence of local and/or diffuse leakage in fundus fluorescein angiography (FFA) and indocyanine green angiography (ICGA), which are important diagnostic methods for CSCR.^5,10,11,12,13^ Due to the multifactorial and complex mechanism of CSCR pathophysiology, several treatment options, such as conventional laser (CL) and verteporfin photodynamic therapy (PDT) have been tried, particularly in the treatment of the chronic type; however, CL was reported to have no significant effect on the final visual acuity or recurrence rate and to have toxic effect on the RPE and photoreceptors.^14,15^ Although successful results were obtained with the standard protocol (full-dose, full-fluence) PDT (SP-PDT), this treatment was also observed to have toxic effects on the RPE and photoreceptors.^16,17,18^ The adverse effects of CL and SP-PDT have prompted studies in recent years on the safety and efficacy of subthreshold micropulse laser (SML), verteporfin PDT with different parameters (half-dose [HD] or half-fluence [HF]), glucocorticoid antagonists, mineralocorticoid receptor (MR) antagonists, and anti-VEGF agents (Figure 1).^19,20,21,22^

This review evaluated current treatment approaches to chronic CSCR based on randomized and nonrandomized studies that accepted symptom duration of at least 3 months as chronic disease and included at least a case series (more than 3 cases).

## Treatment Options

### Subthreshold Micropulse Diode and Yellow Laser

Although it has long been used in the treatment of CSCR, the permanent RPE damage and scarring caused by CL led to the adoption of SML, which minimizes RPE damage with repetitive short pulses (0.1-0.2 ms) that allow the use of less energy. This feature of EML enables the laser to be applied to areas much closer to the fovea.

One drawback of applying SML with repetitive short pulses (0.1-0.2 ms) was that the laser burns were too faint to see with the eye. Ricci et al.^23^ claimed that this problem could be eliminated by applying micropulse diode laser under ICGA guidance to directly visualize the affected area.

In their prospective interventional study, Chen et al.^24^ observed a visual acuity increase of 3 or more letters in 15 of 26 eyes with chronic CSCR that had leakage in the juxtafoveal area and underwent SML therapy (810-nm diode laser), while 5 of the 11 eyes with widespread juxtafoveal RPE leakage required rescue PDT for subretinal fluid resorption. Similarly, Lanzetta et al.^25^ observed subretinal fluid resorption at 1 month in 65% and at the end of the follow-up in 75% of 24 eyes treated with SML (810-nm diode laser) and followed for an average of 14 months. Abd Elhamid^26^achieved subretinal fluid resorption after treatment in 73% of 15 eyes with CSCR treated with SML (577-nm yellow laser). In addition, the authors specifically noted that in 9 cases, the leakage was in foveal avascular zone. 

Of the comparative studies conducted to date, Scholz et al.^27^ applied SML (577-nm yellow laser) to 42 eyes and HD verteporfin PDT (HD-PDT) to 58 eyes diagnosed with chronic CSCR and reported subretinal fluid resorption in 36% of the eyes subjected to SML and 21% of the eyes subjected to PDT at 6 weeks, which was not a statistically significant difference. 

In contrast, Maruko et al.^28^ treated 29 eyes with CSCR and typical focal leakage persisting more than 3 months, 15 with CL and 14 with SML (577-nm yellow laser), and compared their efficacy in terms of complete subretinal fluid resorption and their safety in terms of RPE damage assessed by fundus autofluorescence imaging. Their results showed no significant difference in efficacy between CL and SML (66.7% vs. 64.3%, respectively). However, RPE damage was observed in all eyes with successful outcomes after CL therapy but only in one eye treated successfully with SML. Their study highlighted that SML was at least as efficient as CL and much safer than CL in terms of RPE damage. The authors also stated that their higher success rates compared to the study by Scholz et al.^27^ may be attributed to their exclusion of cases with diffuse leakage from the study. In a comparative study by Özmert et al.^29^, no statistically significant difference in rates of complete resorption of subretinal fluid was observed between HF-PDT in 18 eyes and SML (577-nm yellow laser) therapy in 15 eyes with chronic CSCR (72.2% vs. 80%, respectively). In a comparative, controlled prospective study by Koss et al.^20^, SML (810-nm diode laser) therapy was performed on 16 eyes and intravitreal bevacizumab treatment was performed on 10 eyes with chronic CSCR, and 26 eyes were followed as a control group. The highest rate of complete subretinal fluid resorption at 10 months post-treatment was observed in the SML group, followed by the bevacizumab group, and the differences were statistically significant (SML vs. bevacizumab vs. control: 87.5% vs. 40% vs. 8%). A summary of studies on SML in chronic CSCR is presented in Table 1.

### Intravitreal Anti-VEGF Therapy

Although it is known that choroidal neovascularization (CNV) is not a primary factor in the pathophysiology of CSCR,^30,31^ some authors argue that anti-VEGF agents, which are the popular and effective options for treating CNV, may be effective in resolving the disease by reducing pooling and hyperpermeability in the choroidal vessels.^21,32,33^ There is only one randomized controlled study on the efficacy of anti-VEGF in chronic CSCR in the literature. In this study, performed in Turkey by Artunay et al.^22^, 15 eyes with a history of CSCR persisting longer than 3 months were treated with the anti-VEGF agent bevacizumab and 15 eyes were followed for 6 months without any intervention. They reported complete resorption of subretinal fluid in 80% (n=12) of the treated eyes and 53.3% (n=8) of the untreated eyes (p<0.01). Furthermore, visual acuity was unchanged or improved in all treated eyes and 10 eyes in the follow-up group (p<0.01). 

In one of the nonrandomized, prospective comparative studies, Kim et al.^34^ treated 30 eyes with chronic CSCR with bevacizumab. The researchers grouped eyes that did not respond to the first three injections as anti-VEGF-resistant and the eyes that responded as anti-VEGF-sensitive. Compared to the treatment-resistant group, the treatment-sensitive group showed greater subfoveal choroidal thickness and more choroidal vessel dilation in ICGA before treatment and greater reduction in choroidal thickness after treatment. Based on these findings, the authors noted the importance of the ability to predict response to anti-VEGF therapy before treatment based on subfoveal choroidal thickness and hyperpermeability. In addition to this information, Yannuzzi^35^ stated that the presence of fibrin observed in the fovea on fundus examination indicates leakage from abnormal choroidal vessels and emphasized that PDT in such cases can cause severe RPE damage due to excessive energy accumulation over the fibrin structure. In light of this, anti-VEGF agents may be a better treatment option in terms of preventing potential complications in patients with subretinal fibrin accumulation. A recent meta-analysis of studies concerning anti-VEGF therapy in CSCR resulted in several recommendations. 

Recommended indications for anti-VEGF in chronic CSCR^2,36,37^

1. Patients with subfoveal fibrin accumulation in which focal laser or PDT can be inconvenient

2. When CSCR is complicated by CNV

## Corticosteroid Antagonists

### 1. Glucocorticoid Antagonists

Elevated serum cortisol levels in CSCR patients have been demonstrated previously.^9,38^ Therefore, investigation began into the efficacy of anti-glucocorticoids such as ketoconazole, mifepristone, and finasteride, though only as case series.^39,40^  

Some studies have also demonstrated elevated testosterone levels in CSCR.^41,42^ This information prompted research into the therapeutic efficacy of finasteride, an inhibitor of 5-reductase, an enzyme that is involved in the synthesis of the hormone dihydrotestosterone (which is more potent than testosterone). In a comprehensive study on the efficacy of finasteride, Moisseiev et al.^43^ administered 5 mg/day oral finasteride to 23 patients diagnosed with chronic CSCR (>3 months). After a mean follow-up time of 14.7 months, complete resolution was observed in 75.9% of the patients, while 37.5% had recurrence after discontinuing treatment. However, studies conducted with glucocorticoid antagonists were not randomized or controlled, and therefore, there is still no reliable information on the efficacy of this class of drugs.

### 2. Mineralocorticoid Receptor Antagonists

Several studies have demonstrated that glucocorticoids and mineralocorticoids are co-expressed in the retinal Müller cells and choroidal vessels. With higher circulating levels, these hormones bind to glucocorticoid receptors (GR) and MRs and cause alterations in retinal and choroidal homeostasis, which is considered the most likely factor in the pathophysiology of CSCR.^44,45^ In a randomized controlled comparative study of MR antagonists, Pichi et al.^46^ established 3 groups of 20 patients with chronic CSCR (average duration of 8 months) and administered oral spironolactone to group 1, oral eplerenone to group 2, and placebo to group 3 for the first month. For the second month, they gave eplerenone to group 1 and spironolactone to groups 2 and 3, then discontinued treatment and followed the patients for 2 more months. The authors reported that spironolactone was statistically superior in terms of visual acuity gain and subretinal fluid resolution. They attributed this difference to eplerenone having a 20-fold lower affinity for MR; however, in comparison of adverse effect profiles, they stated that eplerenone exhibits fewer progestinic effects because of its selectivity for MR. The results of other studies of MR antagonists are presented in Table 2. In summary, the MR antagonists spironolactone and eplerenone can be effective options in the treatment of CSCR. However, conducting more randomized controlled studies with these drugs will provide more reliable information regarding both treatment efficiency and adverse effect profile.

## Verteporfin-Photodynamic Treatment

### 1. SP-PDT

The known limitations of argon laser therapy in CSCR and the roles of choroidal vessel dilation and hyperpermeability in CSCR pathophysiology have led to investigation of the efficacy and safety of verteporfin PDT, which was previously proven effective in wet AMD patients (TAP protocol),^47^ in the treatment of CSCR. In the first trial evaluating the efficacy and safety of SP-PDT, carried out by Yannuzzi et al.^48^ subretinal fluid resorption was observed in 60% of 20 chronic CSCR patients after a mean of 6 months. In a study by Cardillo et al.^49^ in which 20 eyes with chronic CSCR were treated with SP-PDT, vision improved in 6 eyes and was unchanged in 14 eyes after an average follow-up period of 12 months, and 81% of the eyes showed complete resorption of subretinal fluid.

Ruiz-Moreno et al.^18^ performed SP-PDT in 82 eyes with chronic CSCR and observed complete resorption of subretinal fluid in all eyes and a statistically significant increase in mean visual acuity (1.9±2.4 Snellen lines) after an average follow-up period of 12 months. In the same study, reactivation (recurring NSD) occurred in 2 eyes, CNV secondary to treatment in 2 eyes (2%), and reactive RPE hyperplasia in 9 eyes (10%). In a study including a total of 42 eyes with chronic CSCR, Reibaldi et al.^16^ treated 19 with SP-PDT and 23 with HF-PDT and reported juxtafoveal CNV at 3 months in only 1 eye (2%) in the SP-PDT group. A summary of studies on SP-PDT in chronic CSCR is presented in Table 3.

### 2. HD-PDT and HF-PDT

Adverse effects such as focal RPE losses, CNV secondary to treatment, chronic choroidal hypoperfusion, and pigmentary changes observed after SP-PDT with verteporfin prompted clinicians to consider modifying the standard treatment protocol. 

Of the clinical studies in the literature investigating the efficacy of verteporfin-PDT in CSCR, only two were done in chronic cases. Bae et al.^50^ randomized 16 eyes with CSCR into two equal groups and treated one group with HF-PDT and the other with intravitreal ranibizumab injection (consecutive monthly injections); at the end of a 3-month follow-up period, they observed complete resorption of subretinal fluid in 6 eyes (75%) in the PDT group and 2 eyes (25%) in the injection group. In the same study, 4 eyes with incomplete resorption after ranibizumab injection underwent rescue HF-PDT and 2 of them showed complete resorption of subretinal fluid. No complications occurred in either group. In another randomized controlled study, Semeraro et al.^51^ gave intravitreal bevacizumab (1.25 mg) injections to 12 eyes and performed HF-PDT in 10 eyes diagnosed with CSCR persisting for an average of more than 3 months, with 9 months of follow-up. At the end of the follow-up period, there were no statistically significant differences between the two groups in terms of mean central macular thickness or change in visual acuity. However, because the number of eyes with complete subretinal fluid resorption was not reported in that study, their results could not be compared in detail with those of other studies. There were also no complications secondary to treatment reported in either group in that study. 

Among the studies on HD-PDT and HF-PDT, Chan et al.^19^ treated 48 eyes with chronic CSCR with HD-PDT (3 mg/m^2^ verteporfin) and reported complete resorption of subretinal fluid in all eyes after 12 months of follow-up and recurrence in 4 eyes (8.3%). Mean visual acuity of the patients increased by 2 lines. No complications occurred in any of the eyes in their study. In another study, Nicolo et al.^52^ performed HD-PDT on 38 eyes with chronic CSCR and observed complete resorption of subretinal fluid in all eyes and recurrence in 5 eyes (13.2%) after a mean follow-up of 14.2 months, and no complications were reported. Senturk et al.^53^ performed HD-PDT on 24 eyes with chronic CSCR and reported complete resorption of subretinal fluid in all of the eyes at 6 months and emphasized that no complications occurred in any of the eyes. 

Of the studies comparing SP-PDT and PDT with different parameters, Reibaldi et al.^16^ treated 42 eyes with chronic CSCR with SP-PDT or HF-PDT. At a mean of 12 months, they reported complete resorption of subretinal fluid in 79% of the eyes treated with SP-PDT and 91% of the eyes treated with HF-PDT. In the SP-PDT group they also observed new atrophy in the treated area on FFA in 1 eye (5%) and juxtafoveal CNV in 1 eye (5%) at 12 months post-treatment. A summary of studies on HD-PDT and HF-PDT in chronic CSCR is presented in Table 4.

In conclusion, publications on PDT in CSCR are still at the level of case series and nonrandomized comparative studies. Randomized controlled clinical trials with much larger samples are needed to evaluate the efficacy and safety of this therapy. A systematic review by Erikitola et al.^54^ in 2014 evaluated results concerning SP-PDT, HD-PDT, and HF-PDT from randomized controlled studies and qualitative observational studies that met at least 70% of the STROBE (Strengthening the Reporting of Observational Studies in Epidemiology) criteria (Table 5).^55^ They concluded that of various parameters, HD-PDT was the treatment option with the lowest adverse event and recurrence rates. In 4 (42.9%) of 7 studies on HD-PDT, complete resorption of subretinal fluid was observed with no recurrence in any of the eyes.^56,57,58,59^ The overall recurrence rate of CSCR in the review varied between 3%-24%, though it was noted that these results were obtained from studies with small sample sizes.

## Conclusion

An evaluation of the literature data regarding current treatment options for chronic CSCR, such as SML, anti-VEGF, MR antagonists, and PDT, suggests that SML is superior to CL in terms of adverse effects and comparable to PDT in terms of efficacy. Assessing the effectiveness of SML using longer-term follow-up data will provide more reliable information for comparison with the effectiveness of PDT. In addition, similar to CL, the ineffectiveness of SML in diffuse RPE leakages is considered an additional disadvantage. Although the valuable prospective randomized study by Artunay et al.^22^ offered promising results, studies on anti-VEGF have usually been reports of a few cases, which limits the power of these studies. Therefore, performing randomized studies with larger sample sizes will yield more reliable results. Moreover, the most probable pathogenesis of the disease is not closely related to the mechanism of action of anti-VEGF, which suggests that these agents may not be very effective. Studies on MR antagonists have shown that these are effective treatment options; however, the results indicate that these short acting agents are more disadvantageous in terms of patient compliance and in comparison with treatment options with more permanent effects such as PDT and SML. Studies with longer follow-up will also provide more definitive data regarding the effectiveness of MR antagonists. Finally, although PDT is known to be more costly than CL, studies indicate that verteporfin PDT is superior to and safer than CL therapy in terms of effectiveness and adverse event profiles, particularly in chronic, subfoveal, and juxtafoveal involvement. In particular, the fact that PDT at different parameters (HD-PDT, HF-PDT) minimized adverse effects such as choroidal ischemia and CNV supports this treatment as an effective and safe treatment option for chronic CSCR.

## Figures and Tables

**Table 1:**
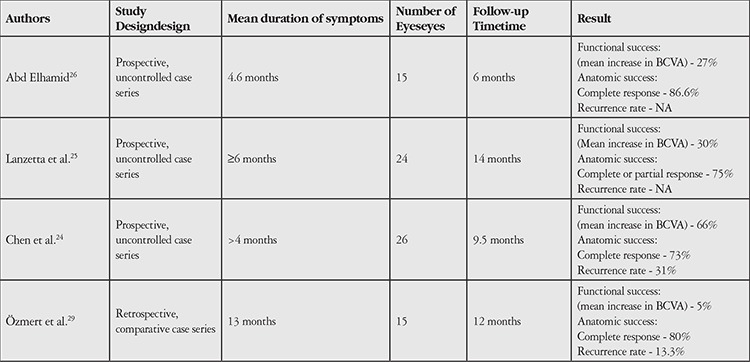
Major studies of subthreshold micropulse laser in the treatment of central serous chorioretinopathy

**Table 2:**
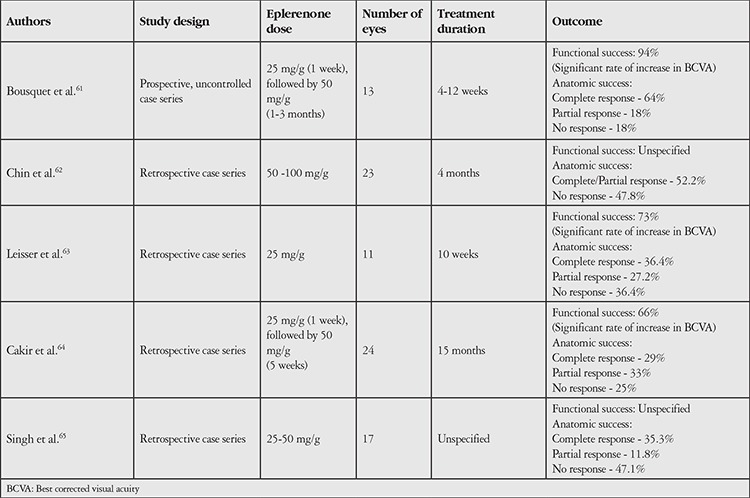
Major studies on mineralocorticoid receptor antagonist therapy (eplerenone) in central serous chorioretinopathy

**Table 3:**
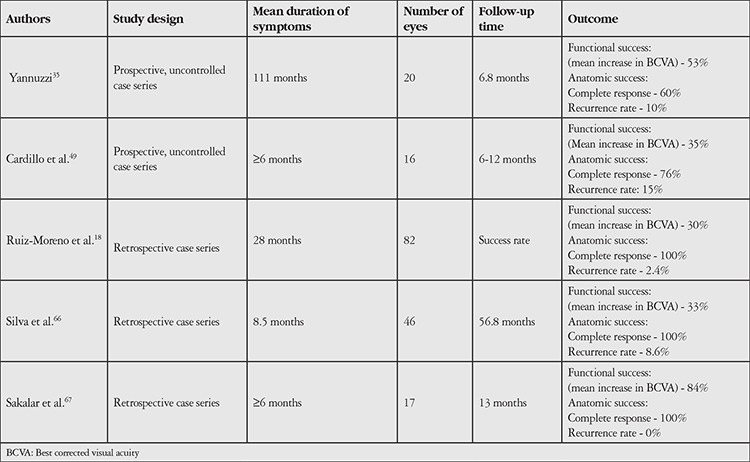
Major studies on standard-protocol verteporfin-photodynamic therapy in central serous chorioretinopathy

**Table 4:**
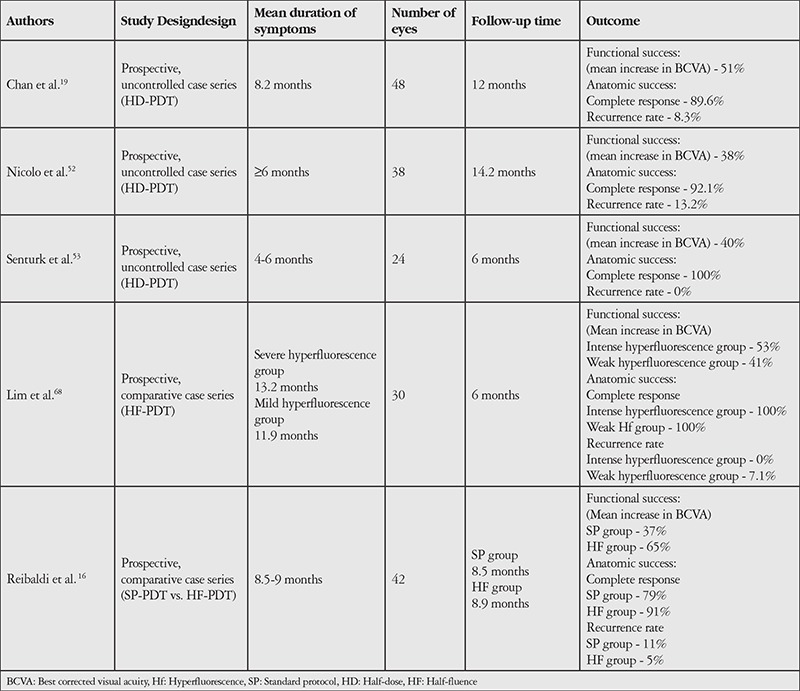
Major studies on half-dose and half-fluence verteporfin–photodynamic therapy in central serous chorioretinopathy

**Table 5:**
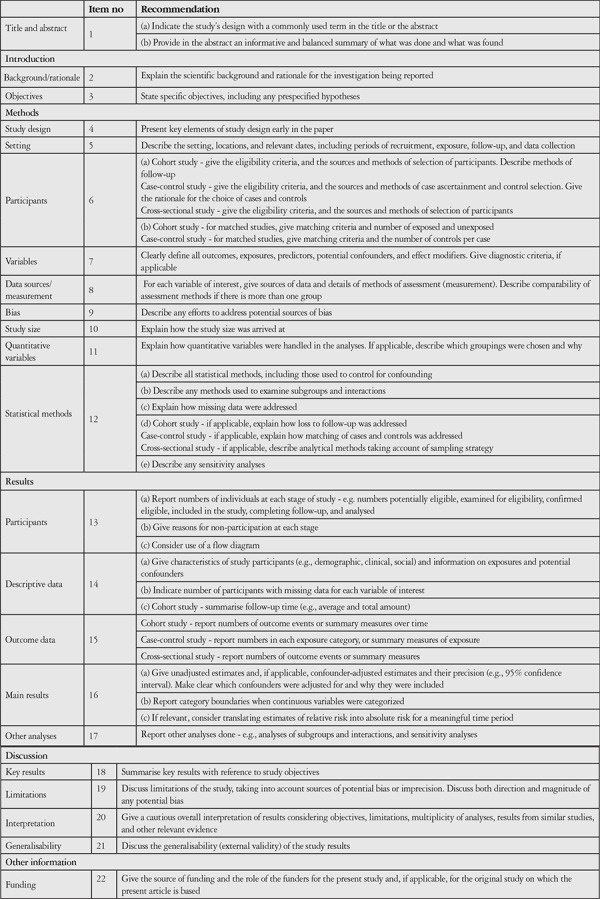
Strengthening the Reporting of Observational Studies in Epidemiology (STROBE) criteria^47,54,55,60^
